# The Systematic Doctor*Read before the Brazos Valley Medical Association, at its eleventh semiannual meeting, at Casimer’s Opera House, Calvert, Texas, May 14 and 15, 1901.

**Published:** 1901-10

**Authors:** 


### TEXAS MEDICAL JOURNAL.

ESTABLISHED JULY, 1885.

PUBLISHED MONTHLY.—SUBSCRIPTION $1.00 A YEAR.

VOL. XVII. AUSTIN, OCTOBER, 1901. No. 4.

### Original Contributions.

For Texas Medical Journal.

### The Systematic Doctor.*

INTRODUCTION.

We physicians, as a class, have a reputation for business laxity, which has become so matter of course in public opinion, as to appreciably limit our incomes as well as our usefulness. It is in the hope that we may receive practical benefit from the discussion of this unsatisfactory condition, that I beg to distract your attention, for the time, from scientific channels, and review some of the principles followed and methods used by numbers of our truly successful brethren in rising above this unfortunate stigma of our profession.

For many years a false ideal, entirely too Utopian for the day and generation, reigned in our midst and still exists to delay that much needed union of business common sense and professional ability. Moreover, the irregularity of the demands on the doctor's time is a constant influence against system, one which he must recognize to combat, and not to be supinely carried by it into a whirlpool of business confusion which cannot but be detrimental to his professional progress. Ours being the most utilitarian of

*Read before the Brazos Valley Medical Association, at its eleventh semi-annual meeting, at Casimer's Opera House, Calvert, Texas, May 14 and 15, 1901.

the professions, the one on which a large share of the world's happiness depends, should most certainly demand and utilize all the benefits to be derived from system. And while these benefits are personal, insuring larger incomes, less work, the elimination of much worry and consequent saving of vitality, they are far from selfish, for the real physician will devote this added time and strength to study and thereby grow in usefulness to his clientele.

RECORDS.

To the earnest doctor, no case is too trivial to be denied record; yet the habit of systematically recording the details of the ever-changing battles against disease is deplorably lacking among us as a class. How many of my hearers have the data from which they can construct the temperature curve of each case of pneumonia, for instance, which they have treated in the last five years, yes, even during the past twelve months; and beside that can refer to each medicament administered in these cases, with effects of same? Most of us are absolutely lacking in the means to tabulate our experience for either personal or general use. Not only are we entirely barren of minutes connected with the more common cases, but many of us are equally innocent of systematic pen scratch in reference to those peculiar and perplexing cases which we would certainly bring before the profession for mutual good if this pernicious laxity was not the rule rather than the exception. I believe that there is a mistaken notion that records mean a lot of trouble. They do mean systematic effort; but it is time spent which brings a rich reward, and if business methods are used it is surprising how little time it actually takes to keep a thoroughly satisfactory record of each case with essential details of temperature, respiration, pulse, excretions, unusual symptoms, treatment, including each dose of medicine given, not neglecting frank statements of diagnostic and therapeutic errors.

How frequently are we perplexed when a patient returns for the prescription which we gave him last year and which "helped the old woman mightily," but of which, alas, we made no record; and being unable to recall the ingredients, cause our patient disappointment, if we are so fortunate as to miss arousing his distrust; or, if he is an up to date business man, his disgust at our slovenly way of conducting our affairs. Repeatedly are we at a loss to remember, at the moment, what is the matter with, and what we have given a patient, who consulted us ten days ago. This is all wrong. The fact that medicine cannot be an exact science is a potent argument that the physician should cultivate precision the more assiduously. Records should be promptly made or you will omit valuable points, and by allowing work to pile up, will become swamped, and your system soon die of suffocation. Notes taken in the presence of the patient, so far from arousing his antagonism, inspire his confidence. He realizes that you are not asking stereotyped questions, but are giving your earnest attention to his case. By the use of the card system, which I will describe, you can make all notes necessary for permanent record in the presence of the patient, in office or at bedside. A practical list of abbreviations will soon come to you. Self-evident words will be omitted, as in a telegram. The essentials of the case will be the more clear to you for the writing. You will always have the details of the case at your finger tips, for your own use, or for exactness in consultation. A decided short cut and a great help in more ways than record keeping is the plan of having a number for each preparation, favorite prescription, tablet, pill and drug used. If, for instance, the scale of 100 is used, it is well to group the remedies into several of the more important therapeutic classes, as anti-periodics, cathartics, expectorants, externals, sedatives, stimulants, tonics, etc., assigning so many numbers to each class. Hence No. 16 might stand for your favorite cathartic, No. 12 for one of your calomel prescriptions, No. 96 for compound syrup of hypophosphites and so on. This plan is a great time saver, and if one is not hopelessly chaotic in his habits, is easily put in operation.

### CHARGES.

We are less open to criticism as to the record we keep of our charges, yet the books of the average doctor would make the gods and bookkeepers weep. Every doctor annually loses hard earned money who does not keep his accounts in such a manner that he can tell at once, not after five to fifteen minutes posting and figuring, the exact amount each patient owes him. By the use of the card system charges are entered at the same time the record is made and that task which every doctor dreads, posting, ceases to be. But whatever your system, make it your rule to have a definite charge for your services, and enter that charge promptly. Never let a patient beat you to your books. It places you at a disadvantage.

Many doctors have drifted into the pernicious habit of discounting their charges when the patient comes to pay. If a man is deserving of charity grant it him freely, give your services gladly to the unfortunate, and enter no charge in your books; but when you do make an entry, do it in the honest belief that you have earned that amount, that it is due you, and do not allow yourself to be cozened out of ten to thirty per cent. of your rightful dues through the weak policy of rebating. If you make any concession of this kind do it in a business way and have it printed on your bills that you allow a certain per cent. for cash within thirty days, but the wisdom of even this plan is questionable.

A certain class of otherwise desirable patients are inclined to argue the point when they settle. They belong to a class termed "kickers" by business men. If you have done your duty in the conduct of the case, and have made your charges with honest consideration, you are in position to be above haggling over a few dollars with an ungrateful or stingy patron. The firm assertion that the amount stated is the amount due unless he considers himself deserving your charity will usually settle the matter. At times it may be advisable to inform a persistent objecter that it is your policy to charge kickers something to kick about. Such a plan will win respect where weak concessions lose it.

### CARD INDEX SYSTEM.

The fact that you seldom find two physicians who keep books alike is proof that the ideal physician's account book has not yet appeared. At least three books are usually necessary to keep things at all straight—a day book, a ledger and some sort of record book. During the last few years business men have found the usefulness of the card index system to be almost unlimited. I am pleased to testify that it is not too good for the doctor. It is a true delight to turn to a patient's card and find the complete record of your services on one side, while on the other is the charge for each corresponding date, with credits entered, and, if you wish, the balance due the first of each month. This seems the acme of condensation. You have made record and charge before the patient has left the office, or, if you wished, have taken the card to the bedside and made the entries there. Your accounts are always ready, they are portable, they are simple. If any question is raised as to a certain charge, simply turning of the card will show the corresponding record. I pass around for illustration the card which I have found to fill all requirements as fully as do some of the more elaborately compiled ones offered by the makers of these systems. Any one desiring to investigate this system further will receive full information by writing to any of the several firms whose advertisements may be seen in all the standard literary and some of the medical periodicals. I can assert that it has made account and record keeping a pleasure where formerly it was dire drudgery. No books whatever are needed unless you wish to keep a simple list of patients and charges in order to show the monthly and yearly amount of practice.

### BILLS AND STATEMENTS.

If I were asked for the one deficiency in our professional relations with the world by which we have been classed so low in the business scale, I would certainly reply that it is our slouchy methods in presenting our accounts. Really, one would think that the average doctor has some doubts as to whether his patient was actually indebted to him, by the way he puts off sending or presenting his bill. It is so unusual to find a physician who follows business principles in this vital matter, and sends out statements monthly, that such an one is the object of wild-eyed surprise. Fie on the annual or semi-annual bill sending habit (the quarterly plan is bad enough save in one crop country districts) ; let "business be business" and give each patient to understand when he opens an account with you that you send out your bills monthly, or, at any rate, quarterly. He will then know what to expect. It is well to have printed on each bill you send out something like the following: "It is my intention to present a statement of all accounts as they appear on my books on the first of each month (charity practice excepted). As long as you are indebted to me and I have confidence in your intention to pay, you will receive a regular monthly statement, unless professional duties interfere or you make an express request to the contrary."

I have found the following notice to bring rich returns when mailed to delayed yearly accounts on the first of each year: "Mr. So and So. Dear Sir: The year closes with a balance of so many dollars of your account with me unpaid. Kindly make satisfactory arrangements regarding same before the fifteenth of January, and oblige, sincerely," etc. Render your bills "For professional services from such a time to date," with an inconspicuous note stating that the items of the account may be seen at the office.

### DEAD BEAT LIST.

Business men and doctors who have business gumption keep a "Dead Beat List." Any man who will receive and ignore twelve monthly, or four quarterly statements, when postal cards cost but one cent, is not deserving further consideration. He already owes you enough. A fifteen cent indexed ledger in which you can enter his name, the date of services and amount due, will save you hundreds of dollars in a few years, if you enter each name with a determination that all future practice for that man is for cash in hand or security, unless he offers satisfactory explanation for his silence. I know a physician who has made his dead beat list a source of profit as well as saving, by refusing to extend further services to any on that list till the old bill with interest to date is paid, and placing all future work on a cash basis. He does not put a name on that list without due consideration, but when it is there his patients understand that he has enough backbone not to be "worked" again. Of course, these methods apply in no wise to the deserving poor. How often do we hear of the "good doctor," who "doesn't seem to manage well." It is our disgrace that those who depend upon us are deprived of so many advantages because we have allowed the world to consider us "easy picking" and have meekly permitted ourselves to be fleeced by those who are not only financially but ethically our debtors.

### OFFICE HOURS.

The general practitioner is prone to overlook the advantages which will accrue to him through the establishment and observance of office hours. It goes without saying that there is a large class of cases which can be most satisfactorily treated in a properly equipped office; in fact, there are many which can be attended to but inefficiently at the patient's home. It is a matter of surprise that so many physicians are willing to simply prescribe for the numerous eye, ear, nose, throat, stomach, genito-urinary and rectal cases which are constantly apply for help. Content to afford a passing relief to a patient that is eager to be cured and who would willingly pay the fee asked by specialists for the local treatment which is necessary for cure. In these days of post-graduate advantages there is no reason why even the doctor who has his home at some cross roads should not have a neat office, a supply of instruments and the ability to treat, not dose, these local conditions. Hundreds of miles of travel will be saved the doctor yearly and as many extra dollars added to his earnings by these means; nor are these more selfish advantages to be compared to the added relief from suffering which he will afford his patients. Every doctor should have at least one hour a day in which he is to be found in his office. Let this be understood and ere long he will have to increase his time in the office. It is a great satisfaction to a patient to know that there is one time in the day when he is sure to find the doctor. How much more professional is this policy than the too common one of spending the unemployed hours lounging about a drug store or swapping yarns on the street. Fidelity to one's office hours is essential. It is well to have them printed on your statements, with the announcement that you will always be found at the office at these times except when called away by cases of sudden or severe illness.

### EXAMINATIONS.

A volume could and should be written on the subject of system in examinations. Many doctors possessing ability multiply mistakes and failures through carelessness or laziness in this vital element in diagnosis. Snap shot work should be relegated to the quackery of which it savors. If a patient is not sufficiently ill to desire a careful investigation of his case, he is not sick enough for physic. But the majority of our clients make an involuntary mental estimate of a physician's ability based upon his thoroughness in examination. Failure to go into a case thoroughly is without excuse. It is a duty the doctor owes to the patient and himself. Without doubt the habit of careful and attentive examinations may become so much an instinctive process that the doctor who drills himself to it becomes in a few years expert, hence rapid. Every physician displays his hall-mark when he examines his patient.

When an emergency call comes one is quite apt, if lacking in system, to find himself at the patient's house unprepared for efficient service. To the modern physician, four articles are requisite for every call: thermometer, hypodermic case, stethoscope and medicine case. If the habit is formed of stopping for a second and recalling these four articles before leaving the office, vexation and possible damage will be avoided. If one is addicted to memonics the following phrase, "The Handmaids of Scientific Medicine," will suggest the four necessary articles. It should be unnecessary to say a word in behalf of the stethoscope in these days of adequate medical education. It is truly the "handmaid" of the careful, discriminating, analytical examiner. I well remember how, in one of my early consultations, a patriarchal brother filled my heart with confusion by bombastically advising me, as I produced my stethoscope to auscultate our patient's heart, "to throw that thing away and cultivate my ear," (*sic*). Poor fellow, his trained and cultivated ear had failed to detect the failing note of that patient's second heart sound, and his cheerful and confident "he'll soon be all right" proved short comfort to the family. I not infrequently see cases in which I give thanks for the foot of free air which the stethoscope vouchsafes during the examination.

STUDY.

In closing, I wish to recall to us all our too often neglected duty to our studies. The modern physician is a student and if he would shun medical old fogyism he must continue to study. And in no branch of his work will the intelligent employment of system prove of more profit. The great Doctor Senn, of Chicago, the peer of living surgeons aand teachers, did not inherit his greatness. His beginnings were humble, but he consecrated himself to his life's work and early formed an unalterable habit of systematic study, and today, in the full fruitage of his marvelous success, he continues to devote the early morning hours to study; and while that vast city, his students and contemporaries lie asleep, Dr. Senn is using those only quiet hours in uninterrupted delving. And when the strenuous activities of the day burst upon him he has already stored his mind with what is new and fresh. Of course he is a leader.

Two quite dissimilar methods for work present themselves to the studious physician. The first is that used by most teachers, and may be termed course study. It consists in taking up a branch and going through it by rote. Devotion to this method makes the theoretical doctor. The second is termed case study; it is, in fact, the essence of clinical instruction. The physician who perfectly utilizes this method makes it a rule to exhaust all available literature before he leaves an obscure case. This method is eminently practical. A little thought will show that while both methods have their advantages the exclusive adoption of either will decidedly limit one's breadth of progress. Undoubtedly the larger proportion of the practitioner's time should be devoted to case study. Woe is he, who will permit either of the extremes, shiftlessness or stress of work, to breed a carelessness to obscure points in his cases. That man's professional growth is completed, the future has but atrophy for him. Nor is the outlook much better for the one who closes his physiology, anatomy and pathology when he receives his diploma. These branches are never mastered, and the progressive doctor will make it his rule to carefully review one of these foundation branches, in the latest work he can obtain on the subject, each year. If this is faithfully done, one's conscience is clear as far as course study is concerned. Case study, if systematically pursued, is the most profitable expenditure of time possible to the active practitioner. It is well to reserve a few pages in your desk or vest pocket memorandum book in which to righteously note every point in any case upon which your knowledge it not clear. Do not let points in anatomy and physiology slip away into oblivion. Look the obscurity up at once, or make a note of it, but keep your note book cleaned up. If an article is worth reading, it is worth understanding. If, for instance, a reference to the pneumogastric wakens a doubt in your mind as to its distribution or function, look it up. A great deal of the value of our journal reading is lost because it is not available for reference. Clippings should be made and filed of all matter which can prove of future use. Your card index system can be used to advantage in preserving reference of all kinds.

This question of study has, I doubt not, caused all of us serious thought, and has been answered by each of us in some way. There are those who have allowed the repeated interruptions of our life's work to degenerate study into fitful snatches of general reference. This is fatal to sustained good work and progress. I insist that it is not a question of can, but of will. There is no doctor with backbone but can arrange his hours so that one of the twenty-four is sacred to study. If Dr. Senn has always made time for two, surely, any of us can secure one. If a record is kept of the points studied during this daily hour's work, one will receive a delightful surprise if at the end of six months he will review these study notes. Ten years of such devotion to duty means a giant stride toward the head of the column.

Success knocks at every man's door once, we are told, but I doubt whether she ever tarried with the man whose hours were disordered and ideals unformed. I trust these hasty references to the more ideal yet essentially practical methods of our business and professional life may stimulate us all to rise above any habits of carelessness, disorder and laxity which may be limiting our usefulness.

